# Adjunctive effects of acupuncture and moxibustion in women with premature ovarian insufficiency receiving hormone replacement therapy: protocol for a multicenter, assessor-blinded, 2 × 2 factorial randomized controlled trial

**DOI:** 10.3389/fendo.2026.1952726

**Published:** 2026-10-19

**Authors:** Chunxia Lu, Tingting Ye, Jin Cui, Bo Chen

**Affiliations:** 1 School of Acupuncture-Moxibustion and Tuina, Guizhou University of Traditional Chinese Medicine, Guiyang, Guizhou, China; 2 Acupuncture and Moxibustion, The First Affiliated Hospital of Guizhou University of Chinese Medicine, Guiyang, Guizhou, China

**Keywords:** acupuncture, factorial design, hormone replacement therapy, moxibustion, Premature ovarian insufficiency, protocol, randomized controlled trial

## Abstract

**Introduction:**

Premature ovarian insufficiency (POI) is characterized by impaired ovarian function before 40 years of age and is associated with menstrual disturbance, infertility, hypoestrogenic symptoms, psychological distress, and long-term health risks. Hormone replacement therapy (HRT) is recommended for symptom control and prevention of estrogen-deficiency-related complications but does not reliably restore ovarian activity or fertility potential. Acupuncture and moxibustion are used as complementary interventions for ovarian dysfunction; however, evidence regarding their independent and combined adjunctive effects in women with POI remains limited.

**Methods:**

This prospective, multicenter, assessor-blinded, 2 × 2 factorial randomized controlled trial will recruit 144 women with POI from three clinical centers. Participants will be randomly assigned in a 1:1:1:1 ratio to estradiol/dydrogesterone alone, acupuncture plus estradiol/dydrogesterone, moxibustion plus estradiol/dydrogesterone, or combined acupuncture and moxibustion plus estradiol/dydrogesterone. The intervention period will last 12 weeks. The primary outcome is the change from baseline to week 12 in antral follicle count (AFC). Secondary outcomes include serum anti-Müllerian hormone (AMH), sex hormones, ovarian hemodynamic parameters, menopausal and anxiety symptoms, Traditional Chinese Medicine syndrome scores, and menstrual status. Clinical pregnancy will be analyzed as an exploratory reproductive outcome during treatment and follow-up at weeks 24 and 48. Factorial models will estimate the main effects of acupuncture and moxibustion, whereas the acupuncture × moxibustion interaction will be considered exploratory.

**Conclusion:**

This trial will evaluate the adjunctive effects of acupuncture and moxibustion on AFC and related secondary clinical outcomes in a selected subgroup of women with POI receiving standard HRT. The factorial design will permit estimation of the independent effects of both interventions and exploratory assessment of their potential interaction.

**Clinical trial number:**

https://itmctr.ccebtcm.org.cn/, identifier ITMCTR2025002408.

## Introduction

Premature ovarian insufficiency (POI) is defined as the loss or substantial decline of ovarian function before the age of 40 years and is typically characterized by menstrual disturbance, elevated gonadotropin levels, hypoestrogenism, and reduced ovarian reserve (1). In addition to infertility, women with POI may experience vasomotor symptoms, sleep disturbance, sexual dysfunction, psychological distress, and impaired quality of life (2). Long-term estrogen deficiency is associated with increased risks of osteoporosis, cardiovascular disease, and other chronic health consequences (3). The etiology of POI is heterogeneous. Pathogenic gene variants account for a proportion of cases (4), autoimmune mechanisms are also recognized (5), and environmental or lifestyle-related factors may contribute in some women (6). Nevertheless, the underlying cause remains unidentified in a substantial proportion of patients (7). POI therefore represents a major challenge in reproductive medicine (8), while its long-term consequences also require sustained multidisciplinary and primary care follow-up (9).

Hormone replacement therapy (HRT) is recommended for women with POI who have no contraindications, primarily to relieve hypoestrogenic symptoms and reduce long-term health risks related to estrogen deficiency (1). Sequential estrogen-progestogen regimens are widely used to provide physiological hormone replacement and endometrial protection (10). In some reproductive clinical settings, estradiol-progestogen therapy may also be used as pretreatment before ovulation induction or *in vitro* fertilization and embryo transfer (IVF-ET), particularly in women with menstrual disturbance and hypoestrogenic status (11). Nevertheless, HRT does not reverse follicular depletion or reliably restore fertility potential (7). For women with POI who wish to conceive using autologous oocytes, limited recruitable follicles and poor ovarian responsiveness remain substantial clinical difficulties (12). Therefore, adjunctive strategies that may improve residual ovarian activity, endocrine regulation, ovarian microenvironment, and reproductive potential are clinically relevant.

Acupuncture and moxibustion are commonly used external therapies in Traditional Chinese Medicine (TCM) for menstrual disorders, infertility, and ovarian dysfunction, and they are included among complementary approaches reported for POI (13). A systematic review and meta-analysis of randomized trials suggested that acupuncture may improve ovarian reserve-related markers, sex hormone profiles, menstrual status, and symptom burden in women with POI (14). An umbrella review likewise reported potential benefit while emphasizing variability in the quality of the underlying evidence (15). A separate systematic review of acupuncture and Chinese herbal medicine also found encouraging but heterogeneous evidence (16). From a biomedical perspective, acupuncture has been proposed to influence reproductive function through modulation of the hypothalamic-pituitary-ovarian axis, autonomic nervous activity, neuroendocrine responses, and pelvic or ovarian blood perfusion (17). Preliminary clinical evidence has reported changes in endocrine parameters, menstrual status, hypoestrogenic symptoms, and psychological distress after acupuncture in women with premature ovarian failure (18). More recent clinical work has also examined phased acupuncture regimens and ovarian-function-related outcomes in POI (19). Moxibustion delivers thermal stimulation to specific acupoints; experimental work suggests effects on oxidative-stress-related pathways and ovarian tissue responses in diminished ovarian reserve (20). Additional animal evidence indicates that moxibustion may influence granulosa-cell apoptosis and PI3K/AKT signaling (21).

From the perspective of TCM, POI is commonly discussed within the categories of “amenorrhea,” “infertility,” and related deficiency syndromes. Kidney deficiency and blood stasis are frequently described as syndrome patterns and therapeutic targets in TCM-based POI studies (16). The acupoints selected in this trial, including CV4, CV6, ST25, ST29, EX-CA1, ST36, SP6, LR3, BL23, BL32, and KI3, are intended to support kidney-related regulation, regulate the Chong and Ren meridians, nourish qi and blood, and improve pelvic circulation from the perspective of TCM. A complex-network analysis of acupuncture and moxibustion prescriptions for diminished ovarian reserve and premature ovarian failure identified CV4, SP6, ST36, BL23, and EX-CA1 among the most frequently used acupoints (22). The present prescription also reflects previous clinical use of Ren-regulating and kidney-tonifying acupuncture combined with Western medicine in POI (23). Using the same predefined prescription across the acupuncture, moxibustion, and combined-treatment groups allows the stimulation modality to vary while the therapeutic framework remains consistent.

Despite increasing clinical interest, several evidence gaps remain. First, existing evidence on complementary and alternative therapies for POI has mainly summarized acupuncture, moxibustion, Chinese medicine, and related approaches, but rigorous trials evaluating acupuncture and moxibustion as adjuncts to standard HRT remain limited (13). Second, the relative effects of acupuncture and moxibustion remain unclear. Third, it is unknown whether combining acupuncture and moxibustion produces additive or synergistic effects beyond either modality alone. To address these gaps, we designed a multicenter, assessor-blinded, 2 × 2 factorial randomized controlled trial. All participants will receive standard estradiol/dydrogesterone therapy, and the two experimental factors will be acupuncture and moxibustion. This design enables estimation of the independent effects of acupuncture and moxibustion and exploration of their interaction. Unlike conventional three-arm or combined-treatment comparisons, this factorial design allows the independent contribution of acupuncture and moxibustion to be estimated while minimizing ambiguity regarding whether potential benefits arise from one modality or their combination. Therefore, the four-group structure was selected to address the clinically relevant question of whether acupuncture and moxibustion provide independent or additional effects when added to standard HRT.

## Methods and analysis

### Study design

This is a prospective, multicenter, assessor-blinded, 2 × 2 factorial randomized controlled trial. Eligible women with premature ovarian insufficiency (POI) will be recruited from three clinical centers and randomly assigned in a 1:1:1:1 ratio to one of four treatment combinations: estradiol/dydrogesterone tablets (Femoston) alone, acupuncture plus Femoston, moxibustion plus Femoston, or combined acupuncture and moxibustion plus Femoston.

The two intervention factors are acupuncture and moxibustion. This factorial design allows estimation of the main effect of acupuncture, the main effect of moxibustion, and the interaction between acupuncture and moxibustion. The intervention period will last 12 weeks, corresponding to three 28-day hormone replacement therapy (HRT) cycles and 36 planned acupuncture and/or moxibustion sessions. The study flow diagram is illustrated in **Figure 1**. The 12-week duration was selected on both empirical and design grounds. In a randomized clinical study of women with premature ovarian insufficiency (POI), a three-month estradiol/dydrogesterone regimen with or without adjunct acupuncture was evaluated, and post-treatment increases in both anti-Müllerian hormone (AMH) and antral follicle count (AFC) were reported; between-group differences in AMH and AFC were also reported (23). This provides POI-specific clinical evidence that these ovarian-reserve markers can show measurable change over a treatment interval comparable to 12 weeks. More directly, a recently published randomized sham-controlled trial in women with diminished ovarian reserve (DOR) used acupuncture three times weekly for 12 weeks and assessed AFC and AMH at week 12. The acupuncture group showed a significantly greater improvement in AFC than the sham group, whereas the between-group difference in AMH was not statistically significant (24). Although DOR and POI are distinct clinical populations, these data provide direct empirical support that AFC can show measurable change over a 12-week acupuncture treatment window and that AMH can be evaluated over the same interval. Comparable 12-week assessment windows have also been prespecified in randomized acupuncture protocols for DOR (25, 26), and the selected duration is consistent with published clinical experience using acupuncture and moxibustion for POI (27) and local experience of combined acupuncture-moxibustion treatment in infertility associated with DOR (28). Taken together, these data support 12 weeks as a scientifically reasonable short-term window for detecting potential changes in AFC and supportive ovarian-function-related measures such as AMH. Importantly, the protocol does not assume that a biomarker change observed at 12 weeks will necessarily be clinically meaningful or represent restoration of the ovarian follicular pool or fertility; the magnitude, precision, and clinical relevance of any observed AFC or AMH change will be interpreted cautiously. Follow-up at weeks 24 and 48 will focus primarily on pregnancy-related outcomes, subsequent fertility treatment, and safety information rather than repeated measurement of ovarian reserve biomarkers. .

**Figure 1:**
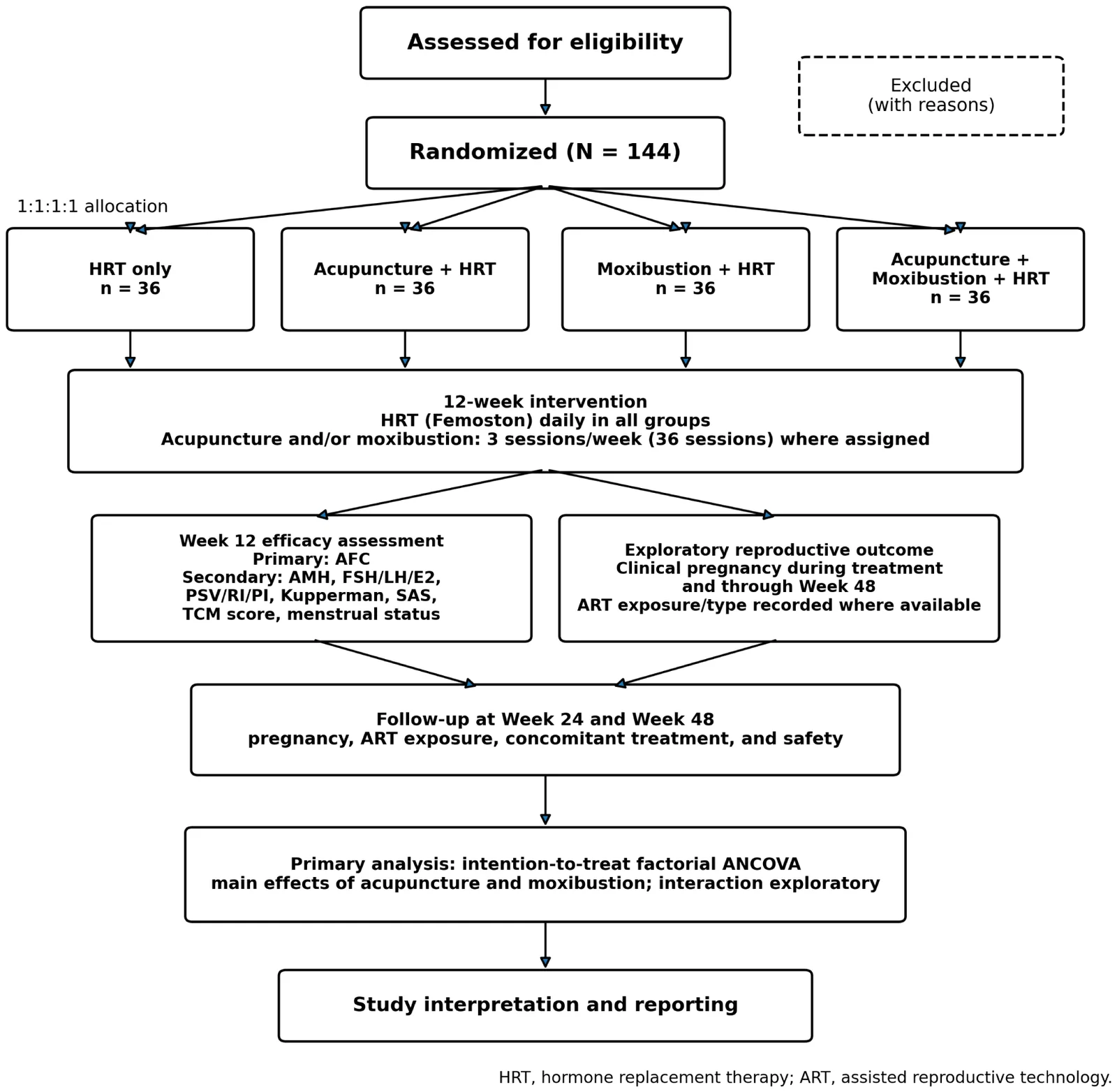
Trial flowchart. Flow diagram of participant recruitment, eligibility screening, 1:1:1:1 random allocation to the four factorial treatment combinations, 12-week intervention, week-12 efficacy assessment (primary outcome: AFC), exploratory reproductive follow-up, and intention-to-treat factorial analysis. AFC, antral follicle count; AMH, anti-Müllerian hormone; HRT, hormone replacement therapy; FSH, follicle-stimulating hormone; LH, luteinizing hormone; E2, estradiol; PSV, peak systolic velocity; RI, resistance index; PI, pulsatility index; SAS, Self-Rating Anxiety Scale; TCM, Traditional Chinese Medicine; ART, assisted reproductive technology; ANCOVA, analysis of covariance.

The trial will be conducted in accordance with the Declaration of Helsinki, Good Clinical Practice principles, the Standard Protocol Items: Recommendations for Interventional Trials (SPIRIT) 2013 recommendations for interventional trial protocols (29), and the Standards for Reporting Interventions in Clinical Trials of Acupuncture (STRICTA) recommendations (30). The procedures for acupuncture, moxibustion, and combined acupuncture and moxibustion are illustrated in **Figure 2**, the Femoston regimen is summarized in **Table 1**, the acupoint selection and manipulation details are presented in **Table 2**, and the schedule of enrollment, interventions, and assessments is presented in **Table 3**.

**Figure 2:**
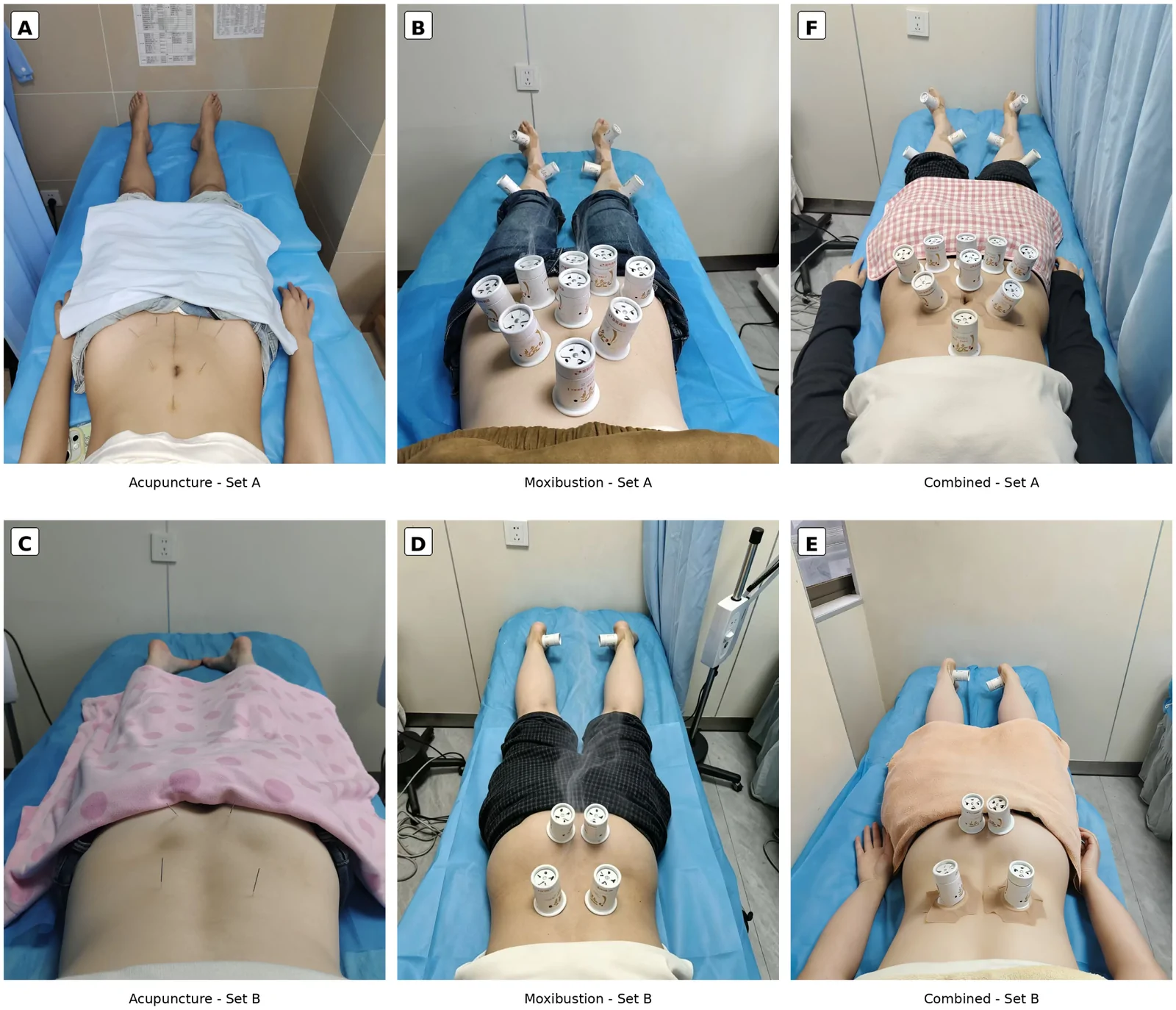
Six-panel representative photographs of the intervention procedures, arranged by acupoint set for direct comparison. First row, Acupoint set A: **(A)** acupuncture group, set A; **(B)** moxibustion group, set A; **(F)** combined acupuncture-moxibustion group, set A. Second row, Acupoint set B: **(C)** acupuncture group, set B; **(D)** moxibustion group, set B; **(E)** combined acupuncture-moxibustion group, set B. Acupoint set A comprises CV12 (Zhongwan), bilateral ST25 (Tianshu), CV6 (Qihai), CV4 (Guanyuan), bilateral ST29 (Guilai), bilateral EX-CA1 (Zigong), bilateral ST36 (Zusanli), bilateral SP6 (Sanyinjiao), and bilateral LR3 (Taichong). Acupoint set B comprises bilateral BL23 (Shenshu), bilateral BL32 (Ciliao), and bilateral KI3 (Taixi). The two sets are applied on successive treatment days, starting with set A, and only one set is used per session. In the combined group, moxibustion is applied during needle retention after Deqi is achieved. Standardized anatomical locations and manipulation details are provided in **Table 2**.

**Table 1:** Regimen of estradiol/dydrogesterone tablets (Femoston).

Menstrual cycle	Tablet composition	Dosage
Days 1-14	White tablet containing estradiol 1 mg	1 tablet once daily
Days 15-28	Grey tablet containing estradiol 1 mg plus dydrogesterone 10 mg	1 tablet once daily

Femoston refers to estradiol/dydrogesterone tablets (Abbott Biologicals B.V., approval number H20150346). Participants will be instructed to take the tablets at the same time each day.

**Table 2:** Acupoint sets, anatomical locations, and manipulation methods used in the acupuncture and moxibustion groups.

Acupoint	Location	Manipulation
Acupoint set A (supine position)		
CV12 (Zhongwan)	On the anterior midline of the upper abdomen, 4 cun above the umbilicus	Perpendicular insertion to a depth of 30–45 mm
ST25 (Tianshu), bilateral	On the abdomen, 2 cun lateral to the umbilicus	Perpendicular insertion to a depth of 30–45 mm
CV6 (Qihai)	On the anterior midline of the lower abdomen, 1.5 cun below the umbilicus	Perpendicular insertion to a depth of 30–45 mm
CV4 (Guanyuan)	On the anterior midline of the lower abdomen, 3 cun below the umbilicus	Perpendicular insertion to a depth of 30–45 mm
ST29 (Guilai), bilateral	On the lower abdomen, 4 cun below the umbilicus and 2 cun lateral to the anterior midline	Perpendicular insertion to a depth of 25–30 mm
EX-CA1 (Zigong), bilateral	On the lower abdomen, 4 cun below the umbilicus and 3 cun lateral to the anterior midline	Perpendicular insertion to a depth of 25–30 mm
ST36 (Zusanli), bilateral	On the anterolateral aspect of the leg, 3 cun below ST35 and one finger-breadth lateral to the anterior crest of the tibia	Perpendicular insertion to a depth of 30–45 mm
SP6 (Sanyinjiao), bilateral	On the medial aspect of the leg, 3 cun above the tip of the medial malleolus, posterior to the medial border of the tibia	Perpendicular insertion to a depth of 30–45 mm
LR3 (Taichong), bilateral	On the dorsum of the foot, in the depression anterior to the junction of the first and second metatarsal bones	Perpendicular insertion to a depth of 15–20 mm
Acupoint set B (prone position)		
BL23 (Shenshu), bilateral	In the lumbar region, 1.5 cun lateral to the lower border of the spinous process of the second lumbar vertebra	Perpendicular insertion to a depth of 15–30 mm
BL32 (Ciliao), bilateral	In the sacral region, in the second posterior sacral foramen	Oblique insertion toward the second posterior sacral foramen to a depth of 60–75 mm
KI3 (Taixi), bilateral	On the medial aspect of the foot, in the depression between the tip of the medial malleolus and the Achilles tendon	Perpendicular insertion to a depth of 15–20 mm

Acupoint localization follows the national standard nomenclature and location of acupuncture points and the textbook Acupuncture and Moxibustion (2021 edition). The same acupoint prescription is used in the acupuncture, moxibustion, and combined acupuncture and moxibustion groups. Acupoint set A is performed in the supine position and acupoint set B in the prone position; the sets are alternated on successive treatment days, starting with set A, and only one set is used per session. For the moxibustion group, the same acupoints are used without needle insertion. Cun is a traditional proportional unit used for acupoint location.

**Table 3:** Schedule of enrollment, interventions, and assessments.

Item	Screening	Baseline	Intervention period	End of treatment	Follow-up (Week 24)	Follow-up (Week 48)
	Week −4 to 0	Week 0	Weeks 1–12	Week 12	Week 24	Week 48
Enrollment						
Eligibility screening	✓					
Written informed consent	✓					
Demographic information		✓				
Medical and reproductive history		✓				
TCM syndrome assessment		✓		✓		
Randomization		✓				
Interventions						
Estradiol/dydrogesterone tablets (Femoston)			✓			
Acupuncture			✓			
Moxibustion			✓			
Combined acupuncture and moxibustion			✓			
Primary outcome						
Antral follicle count (AFC), total bilateral count		✓		✓		
Secondary outcomes						
Anti-Müllerian hormone (AMH), ng/mL		✓		✓		
Serum sex hormones: FSH/LH (mIU/mL), E2 (pg/mL)		✓		✓		
Ovarian hemodynamic indices: PSV (cm/s), RI and PI (unitless)		✓		✓		
Modified Kupperman Scale		✓		✓		
Self-Rating Anxiety Scale (SAS)		✓		✓		
Menstrual status		✓		✓		
Exploratory clinical pregnancy outcome			✓	✓	✓	✓
Concomitant treatments		✓	✓	✓	✓	✓
Safety outcomes						
Safety assessment			✓	✓	✓	✓
Adverse events			✓	✓	✓	✓
Treatment adherence			✓	✓		

TCM, Traditional Chinese Medicine; AFC, antral follicle count; AMH, anti-Müllerian hormone; FSH, follicle-stimulating hormone; LH, luteinizing hormone; E2, estradiol; PSV, peak systolic velocity; RI, resistance index; PI, pulsatility index; SAS, Self-Rating Anxiety Scale; ART, assisted reproductive technology. AFC, AMH, FSH/LH/E2, ovarian hemodynamic indices, modified Kupperman Scale, SAS, TCM syndrome assessment, and menstrual status are assessed at baseline and week 12 only. For participants with spontaneous menstruation, baseline and week-12 AFC examinations and AMH blood sampling, together with other scheduled blood tests and transvaginal ultrasound examinations, are performed on days 2–4 of the menstrual cycle; participants without spontaneous menstruation are assessed at the prespecified study timepoint without waiting for menstrual onset. Clinical pregnancy and subsequent fertility treatment are recorded during the intervention and at weeks 12, 24, and 48. Adverse events are recorded throughout study participation.

Because acupuncture and moxibustion are physical interventions, participants and treatment providers cannot be blinded. Therefore, this study adopts an assessor-blinded design. Recruitment and eligibility-screening personnel will be separated from the practitioners delivering acupuncture or moxibustion, and treatment practitioners will not participate in outcome assessment. Outcome assessors, ultrasound physicians, laboratory personnel, data-entry personnel, and statisticians will remain blinded to group allocation. Participants will be instructed not to disclose their treatment assignment during assessments, and any accidental unblinding will be documented. The primary endpoint will be assessed at week 12; weeks 24 and 48 will be used for reproductive follow-up and safety/concomitant-treatment documentation.

### Participants

#### Recruitment

Participants will be recruited from the Acupuncture Clinic and Infertility Department of the First Affiliated Hospital of Guizhou University of Traditional Chinese Medicine, the Reproductive Center of the Affiliated Hospital of Guizhou Medical University, and the Reproductive Center of Guizhou Provincial People’s Hospital. Additional recruitment will be conducted through hospital advertisements, outpatient referrals, and online announcements.

Potential participants will undergo preliminary screening by trained research staff. Women who appear eligible will be invited for a screening visit. The study objectives, procedures, potential benefits, risks, confidentiality protections, and the right to withdraw at any time will be explained verbally and in writing. Written informed consent will be obtained before any trial-specific procedures are performed.

#### Diagnostic criteria

The diagnosis of POI will be made according to the European Society of Human Reproduction and Embryology guideline (1) and the Chinese expert consensus on the clinical diagnosis and treatment of premature ovarian insufficiency (31). Participants must meet the following criteria: 1) Age younger than 40 years; 2) Oligomenorrhea or amenorrhea for at least 4 months; 3) Basal serum follicle-stimulating hormone (FSH) >25 U/L on at least two occasions, with an interval of more than 4 weeks; 4) Ultrasound examination excluding organic pelvic lesions and showing no obvious dominant follicle; 5) Exclusion of other congenital or acquired causes of menstrual abnormality, including polycystic ovary syndrome, hyperprolactinemia, and thyroid dysfunction.

#### Traditional Chinese medicine diagnostic criteria

Traditional Chinese Medicine (TCM) diagnosis will be based on standard TCM gynecological diagnostic principles and relevant Chinese clinical guidelines for gynecological disorders and new TCM drug research (16).

The main symptom is amenorrhea or oligomenorrhea for at least 4 months, with or without scanty menstrual flow, pale-dark menstrual blood, or thin menstrual consistency. Secondary symptoms may include hot flashes, night sweats, vaginal dryness, dyspareunia, palpitations, insomnia, vivid dreams, dizziness, tinnitus, fatigue, aversion to cold, cold limbs, lower abdominal cold pain, poor appetite, loose stools, nocturia, decreased libido, irritability, or depression. Tongue and pulse manifestations may include a dark tongue, petechiae or ecchymosis, scanty or white coating, and a deep, thin, wiry, or choppy pulse. The main symptom plus at least one secondary symptom, together with tongue and pulse findings, will support the TCM diagnosis.

#### Inclusion criteria

Participants must meet all of the following criteria: 1) Diagnosis of POI and presence of reproductive needs; 2) Age 22–40 years; 3) Basal FSH between 25 U/L and 40 U/L, confirmed by two tests at least 4 weeks apart; 4) Currently in the pretreatment phase before *in vitro* fertilization and embryo transfer (IVF-ET) and not yet undergoing ovulation induction (11); 5) No use of hormonal medication, acupuncture, moxibustion, or related POI treatment within the previous month; 6) Ability to understand the study procedures and provide written informed consent. These eligibility criteria intentionally define a selected reproductive-age subgroup of women with POI who have reproductive needs and are preparing for assisted reproduction; they do not represent the full clinical spectrum of POI.

#### Exclusion criteria

Participants will be excluded if they meet any of the following criteria: 1) Hyperprolactinemia, thyroid dysfunction, congenital reproductive tract malformation, or other endocrine or gynecological disorders that may affect ovarian function; 2) Severe cardiovascular, cerebrovascular, hepatic, renal, hematopoietic, malignant, or psychiatric disease; 3) Contraindications to estradiol/dydrogesterone tablets, including hypersensitivity to the study medication, known or suspected estrogen-dependent malignancy, unexplained vaginal bleeding, active thromboembolic disease, or severe liver disease, consistent with established HRT safety considerations in POI (10); 4) History of severe needle fainting, severe moxibustion intolerance, or severe allergic constitution; 5) Planned use of other POI-related interventions during the study period; 6) Incomplete clinical information, poor expected compliance, or inability to complete the study schedule.

#### Withdrawal and discontinuation criteria

Participants may withdraw voluntarily at any time without affecting routine clinical care. Trial treatment may be discontinued if any of the following occurs: 1) Inability to tolerate acupuncture or moxibustion; 2) Serious adverse event or adverse reaction requiring discontinuation; 3) Pregnancy during the intervention period; 4) Use of prohibited concomitant treatment; 5) Investigator judgment that continued participation may compromise participant safety.

For participants who withdraw or discontinue treatment, investigators will record the date, reason, last treatment session, available outcome data, and adverse events. Participants who become pregnant will discontinue acupuncture and moxibustion immediately and will be transferred to pregnancy follow-up. Femoston will also be discontinued or adjusted according to obstetric or reproductive specialist advice, and the participant will enter pregnancy follow-up. Pregnancy occurrence and pregnancy outcomes will be retained as clinically important outcome data.

### Randomization and blinding

#### Randomization and allocation concealment

The randomization sequence will be generated using SPSS version 26.0 to produce 144 random numbers and corresponding treatment assignments. Allocation will be performed in a 1:1:1:1 ratio, with 36 participants assigned to each of the four treatment groups. Cards containing the sequential number, random number, and treatment assignment will be placed in sequentially numbered opaque sealed envelopes; the envelope number will correspond to the number on the enclosed allocation card. After eligibility has been confirmed and baseline assessment completed, envelopes will be opened in sequence according to participant enrollment order, and the indicated treatment will be assigned. Outcome assessors and statisticians will not have access to the allocation information during outcome assessment and analysis.

#### Blinding

Due to the inherent nature of acupuncture and moxibustion, participants and treatment providers cannot be blinded. Recruitment and screening personnel will be separate from intervention practitioners, and practitioners will not participate in outcome assessment. Outcome assessors, ultrasound physicians, laboratory technicians, data-entry personnel, and statisticians will remain blinded to group allocation throughout the trial. Participants will be instructed not to disclose their group assignment during assessments. Any accidental unblinding will be documented and reported.

### Interventions

All participants will receive standard oral estradiol/dydrogesterone tablets (Femoston). Participants allocated to the acupuncture, moxibustion, or combined intervention groups will receive the assigned external therapy in addition to Femoston. The intervention period will last 12 weeks, corresponding to three menstrual cycles. An overview of the standardized intervention procedures is provided in **Figure 2**. The Femoston regimen is shown in **Table 1**, and the acupoint selection, locations, and manipulation methods are summarized in **Table 2**.

### Femoston group

Participants in the control group will receive estradiol/dydrogesterone tablets (Femoston, Abbott Biologicals B.V., approval number H20150346), following the regimen recommended by the Chinese expert consensus on POI (31). The detailed dosing schedule is presented in **Table 1**. Each 28-day cycle consists of: 1) Days 1–14: one white tablet containing estradiol 1 mg, once daily; 2) Days 15–28: one grey tablet containing estradiol 1 mg plus dydrogesterone 10 mg, once daily. Participants will be instructed to take the tablets at the same time each day. Missed doses, adverse reactions, and concomitant medication use will be recorded in a patient diary.

### Acupuncture plus Femoston group

Participants will receive the same Femoston regimen plus acupuncture three times per week, once every other day, for 12 weeks, totaling 36 sessions. The predefined acupoint prescription will be divided into two treatment sets according to participant position and operational requirements. Acupoint set A, performed in the supine position, includes CV12 (Zhongwan), bilateral ST25 (Tianshu), CV6 (Qihai), CV4 (Guanyuan), bilateral ST29 (Guilai), bilateral EX-CA1 (Zigong), bilateral ST36 (Zusanli), bilateral SP6 (Sanyinjiao), and bilateral LR3 (Taichong). Acupoint set B, performed in the prone position, includes bilateral BL23 (Shenshu), bilateral BL32 (Ciliao), and bilateral KI3 (Taixi). The prescription is consistent with acupoint combinations previously used in clinical acupuncture studies of POI (23). The two sets will be used alternately on successive treatment days, starting with acupoint set A, and only one set will be used at each session. These sets are treatment schedules rather than additional participant groups. The acupoint selection, anatomical locations, and manipulation methods are summarized in **Table 2**.

Disposable sterile stainless-steel acupuncture needles will be used. According to acupoint location, needles of 0.25 mm × 40 mm and 0.30 mm × 75 mm will be selected. After routine skin disinfection, needles will be inserted according to standardized depths and angles: CV12, ST25, CV6, CV4, ST36, and SP6 will be inserted perpendicularly to a depth of 30–45 mm; ST29 and EX-CA1 will be inserted perpendicularly to a depth of 25–30 mm; BL23 will be inserted perpendicularly to a depth of 15–30 mm; BL32 will be inserted obliquely toward the second posterior sacral foramen to a depth of 60–75 mm; and KI3 and LR3 will be inserted perpendicularly to a depth of 15–20 mm. Gentle lifting, thrusting, and twirling will be applied to elicit Deqi, characterized by soreness, numbness, distension, or heaviness, in accordance with commonly reported acupuncture practice in this field (32). Needles will be retained for 30 minutes. After needle removal, sterile cotton swabs will be used to compress needle sites to prevent bleeding or hematoma.

### Moxibustion plus Femoston group

Participants will receive the same Femoston regimen plus moxibustion at the same predefined acupoints used in the acupuncture group. Acupoint sets A and B will be used alternately on successive treatment days, starting with set A, with one set used per session. The moxibustion arm therefore uses the same anatomical prescription described for acupuncture, allowing the stimulation modality to differ while acupoint selection remains constant. Treatment will be delivered three times per week for 12 weeks, totaling 36 sessions.

Self-adhesive moxa column patches measuring 10 mm × 43 mm will be used. Each moxa column will be approximately 10 mm in diameter and 15 mm in height and will be fixed to an adhesive base with an ash-proof protective cover. After ignition, the moxa patch will be applied vertically to the selected acupoint. The procedure will be adjusted to maintain a comfortable warm sensation without burning pain. If excessive heat is reported, the distance between the moxa column and the skin will be increased or the treatment will be discontinued. Each moxa column burns for approximately 10 minutes, and three moxa columns will be used per session, resulting in a total treatment duration of approximately 30 minutes. Local skin condition and participant tolerance will be monitored throughout the procedure to prevent burns and ensure treatment safety.

### Combined acupuncture and moxibustion plus Femoston group

Participants will receive Femoston plus simultaneous acupuncture and moxibustion at the same acupoints and with the same frequency as described above. Acupuncture will first be performed until Deqi is achieved; moxibustion will then be applied during needle retention. Procedures will be standardized to ensure that the acupuncture and moxibustion components are consistent with those delivered in the single-modality groups.

### Treatment fidelity and practitioner qualification

All acupuncture and moxibustion procedures will be performed by licensed TCM acupuncturists who have received study-specific training. Before trial initiation, all practitioners will undergo unified training on acupoint localization, needle insertion depth, manipulation techniques, moxibustion procedures, adverse event management, and case report form completion.

### Adherence and concomitant treatments

Participants completing at least 80% of the planned treatment sessions will be considered adherent for per-protocol analysis. Missed sessions due to heavy menstruation or other acceptable reasons will be recorded but will not be routinely made up, in order to preserve the prespecified treatment schedule.

During the intervention period, participants will be discouraged from using additional POI-related treatments, including other hormonal therapies, Chinese herbal prescriptions aimed at ovarian function, acupuncture, moxibustion, or other reproductive interventions. Necessary rescue treatment for clinically significant symptom aggravation will be permitted when judged medically necessary and will be recorded as a protocol deviation or concomitant therapy. Use of medication, supplements, or reproductive treatment during follow-up will be documented. Initiation of ovulation induction or IVF-ET during the 12-week intervention will be discouraged unless clinically necessary, and any assisted reproductive treatment during follow-up will be recorded in detail for sensitivity analyses.

### Outcome measurements

The outcomes and assessment timepoints are presented in **Table 3**. Outcome measures were selected to reflect the multidimensional clinical features of POI, including ovarian reserve, endocrine function, ovarian hemodynamics, symptom burden, menstrual status, and reproductive outcomes. The registered primary outcome and the secondary ovarian-function and symptom outcomes will be assessed at baseline and week 12. Clinical pregnancy and subsequent fertility treatment will be documented during the intervention and at follow-up visits at weeks 24 and 48. Adverse events will be recorded throughout study participation.

For participants with spontaneous menstruation, baseline and week-12 AFC ultrasound examinations and AMH blood sampling will be performed on days 2–4 of the menstrual cycle to standardize assessment timing. The same cycle-day window will be used for the accompanying blood tests and transvaginal ultrasound measurements where applicable. For participants without spontaneous menstruation, AFC, AMH, and other scheduled measurements will be performed at the corresponding prespecified study timepoint without waiting for menstrual onset. The timing of each assessment relative to menstrual status will be documented.

#### Primary outcome

The primary outcome is the change from baseline to week 12 in antral follicle count (AFC). AFC was selected as the primary outcome because it provides a direct ultrasound-based estimate of the recruitable antral follicle pool and was the primary indicator specified in the trial registration. AFC is widely used as a marker of ovarian reserve in the clinical evaluation of POI (7). It has also been used as an outcome in randomized acupuncture studies summarized in the POI evidence base (14). In this trial, AFC will therefore be interpreted as a marker of residual ovarian activity rather than a direct measure of fertility.

AFC will be assessed by transvaginal color Doppler ultrasound and reported as the total number of 2–9 mm antral follicles in both ovaries. To minimize inter-center and inter-device measurement variability, all baseline and week-12 AFC examinations, regardless of recruitment center, will be performed centrally in the Department of Ultrasound of the First Affiliated Hospital of Guizhou University of Traditional Chinese Medicine using a GE HealthCare LOGIQ Fortis ultrasound system (software version R4.6.2) equipped with an RIC5-9-D transvaginal volume probe (4.0–9.0 MHz). Antral follicles will be defined as anechoic follicles measuring 2–9 mm in diameter. Ultrasound physicians will receive unified protocol-specific training and will remain blinded to treatment allocation. Whenever feasible, the same blinded physician will examine the same participant at baseline and week 12. Because AFC imaging is centralized rather than performed independently at the three recruitment centers, a separate cross-center inter-observer coefficient is not planned; observer variability will instead be minimized through centralized acquisition, standardized training, and same-examiner reassessment whenever feasible.

#### Secondary outcomes

Secondary outcomes will be assessed at baseline and week 12 unless otherwise specified. They are intended to provide supportive information on ovarian reserve, endocrine status, ovarian perfusion, symptom burden, and menstrual function; they will not be interpreted as confirmatory efficacy endpoints.

Anti-Müllerian hormone (AMH): Serum AMH will be measured in ng/mL at the central laboratory of the First Affiliated Hospital of Guizhou University of Traditional Chinese Medicine using the Elecsys AMH assay (Roche Diagnostics, Switzerland) on a cobas e 602 electrochemiluminescence immunoassay platform. Fasting venous blood samples of 5 mL will be processed within 2 hours of collection; samples will be centrifuged at room temperature at 3000 rpm for 15 minutes, and the separated serum will be aliquoted and stored at −80 °C until analysis. Samples collected at the three recruitment centers will follow the same standardized collection, clotting, centrifugation, aliquoting, and storage procedures and will be transported on dry ice to the central laboratory. Sample identifiers will be verified on receipt, and paired baseline and week-12 samples will be batch analyzed centrally wherever feasible to minimize inter-laboratory and inter-batch variability. AMH provides complementary information on residual follicular activity but will be interpreted as a secondary ovarian reserve marker rather than a direct measure of fertility.

Serum sex hormones: Follicle-stimulating hormone (FSH) and luteinizing hormone (LH) will be reported in mIU/mL and estradiol (E2) in pg/mL. These hormones will be measured using chemiluminescent immunoassays.

Ovarian hemodynamic parameters: Peak systolic velocity (PSV, cm/s), resistance index (RI, unitless), and pulsatility index (PI, unitless) will be measured using transvaginal color Doppler ultrasound. Bilateral ovarian measurements will be obtained and the mean values of both ovaries will be used for analysis.

Modified Kupperman Scale: The modified Kupperman Scale will be used to assess hypoestrogenic and perimenopausal symptom burden, with higher scores indicating greater symptom severity.

Self-Rating Anxiety Scale: The Self-Rating Anxiety Scale (SAS) will be used to assess anxiety-related symptom burden. The standard score will be calculated as the total raw score multiplied by 1.25. Anxiety is included as a secondary patient-reported outcome because psychological distress is an important component of the clinical burden of POI (2).

TCM syndrome score: The Traditional Chinese Medicine (TCM) syndrome score will be used as a secondary symptom-based outcome to evaluate changes in menstrual manifestations, vasomotor symptoms, sleep, fatigue, cold intolerance, abdominal symptoms, mood, tongue characteristics, and pulse manifestations within the TCM assessment framework.

Menstrual status: Menstrual outcomes will include menstrual recovery, cycle length, menstrual volume, and menstrual regularity.

#### Exploratory reproductive outcome

Clinical pregnancy will be considered an exploratory reproductive outcome rather than a confirmatory efficacy endpoint because the trial was not powered primarily to detect differences in pregnancy outcomes. Clinical pregnancy will be defined as the presence of an intrauterine gestational sac with fetal cardiac activity confirmed by ultrasound. Pregnancy events will be recorded during the 12-week intervention and at follow-up assessments at weeks 24 and 48.

For the overall exploratory pregnancy analysis, participants will remain classified according to their randomized treatment allocation. Subsequent fertility treatment, including ovulation induction, IVF-ET, or other assisted reproductive technology (ART), will be documented where available. The presence and type of ART will be considered when interpreting pregnancy outcomes, and pregnancy events will additionally be summarized as spontaneous conceptions or conceptions following ART where this information is available. These post-randomization subgroup summaries will be regarded as exploratory and will not be interpreted as preserving randomized causal comparisons.

#### Safety outcomes

Safety outcomes include the incidence, type, protocol-defined severity grade, duration, management, relatedness, and outcome of all adverse events throughout the trial.

### Safety assessment

An adverse event is defined as any unfavorable medical occurrence during the trial, regardless of whether it is considered related to the study intervention. Adverse events potentially associated with acupuncture include vasovagal reactions, subcutaneous hematoma, needle retention, local pain, infection, and needle breakage. Potential moxibustion-related adverse events include allergic reactions, burns, smoke intolerance, and moxibustion-induced syncope. HRT-related symptoms that may occur during treatment include breast tenderness, nausea, headache, and abnormal vaginal bleeding (10). Other medically relevant events will also be recorded irrespective of presumed causality.

For each adverse event, the onset date, duration, severity, management, outcome, effect on continuation of trial treatment, and the investigator-assessed relationship to acupuncture, moxibustion, or HRT will be recorded. The protocol uses a four-level safety grading scheme: grade 1, no adverse reaction; grade 2, mild adverse reaction requiring no treatment and allowing study treatment to continue; grade 3, moderate adverse reaction requiring treatment but permitting continuation after management; and grade 4, an adverse reaction requiring discontinuation of study treatment. Each observed adverse event will additionally be classified by the investigator as treatment-related or unrelated to the relevant study intervention. Serious adverse events will be reported to the ethics committee within 24 hours, and participants will receive appropriate medical care.

### Data collection and management

All data will be recorded in standardized case report forms by trained research staff. Data will include demographic characteristics, disease history, reproductive history, baseline laboratory results, ultrasound findings, treatment records, adherence, concomitant therapies, outcome assessments, and adverse events. Double data entry will be performed to reduce entry errors. Data consistency checks and range checks will be conducted regularly. Participant confidentiality will be protected by assigning each participant a unique study identification number. Personal identifiers will be stored separately from research data. Only authorized study personnel will have access to identifiable information. Source documents, signed informed consent forms, and completed case report forms will be stored securely at each study site.

### Sample size calculation

This study uses a balanced four-group design within a 2 × 2 factorial framework, with acupuncture and moxibustion as the two randomized intervention factors. AFC, the registered primary outcome, was used for sample-size planning. According to previous literature, post-treatment AFC was 3.97 ± 1.15 in the Femoston-only group and 5.14 ± 1.33 in the acupuncture plus Femoston group (23). The between-group mean difference was 1.17, with a pooled standard deviation of approximately 1.24, corresponding to Cohen’s d of approximately 0.94.

The original sample-size estimation was based on an overall four-group comparison. With a two-sided alpha level of 0.05 and 90% power, G*Power version 3.1 estimated that 26 participants per group would be required. Allowing for an anticipated dropout rate of approximately 20%, at least 33 participants per group were required. The planned sample size was increased to 36 participants per group, resulting in a total sample size of 144 participants.

Within the factorial framework, each level of a randomized factor will include up to 72 participants (72 assigned to acupuncture vs 72 not assigned to acupuncture; likewise for moxibustion). Using the AFC planning benchmark of Cohen’s d = 0.94, a two-sided alpha level of 0.05, and 72 participants per factor level yields greater than 99% power to detect a main effect of that magnitude. Conversely, with 72 participants per factor level, the approximate minimum detectable standardized effect at 90% power is Cohen’s d = 0.54 (approximately 0.47 at 80% power). Because the available d = 0.94 estimate was derived from an acupuncture comparison and no equally robust moxibustion-specific effect estimate was available, these calculations are planning benchmarks and do not imply that the true moxibustion effect is d = 0.94. The study was not powered specifically for the acupuncture × moxibustion interaction, which will therefore be exploratory. The sample size was also not based on pregnancy outcomes.

### Analysis sets

The intention-to-treat (ITT) population will include all randomized participants and will analyze participants according to their originally assigned treatment allocation, regardless of subsequent treatment adherence, treatment discontinuation, or crossover. The ITT population will constitute the primary efficacy analysis population.

The per-protocol (PP) population will include participants who complete the primary outcome assessment, receive at least 80% of their planned acupuncture and/or moxibustion sessions when applicable, and have no major protocol deviations. The 80% threshold is the prespecified adherence criterion for PP analysis rather than, by itself, a definition of a major protocol deviation. Major deviations include receipt of prohibited concomitant POI-related treatment, treatment crossover, or other departures judged to materially compromise interpretation of the primary outcome. PP analyses will be conducted as sensitivity analyses and will not replace the primary ITT analysis.

The safety population will include all participants who receive at least one dose of Femoston or at least one session of their assigned acupuncture and/or moxibustion intervention. Safety analyses will be based on the treatment actually received where appropriate.

### Statistical analysis

All statistical analyses will be performed using SPSS version 26.0 or equivalent validated statistical software by statisticians blinded to treatment allocation. Because recruitment has not yet begun, a detailed statistical analysis plan will be finalized before the first participant is enrolled and will remain locked before database lock and unblinding of treatment codes.

Continuous variables will be summarized as mean and standard deviation when approximately normally distributed and as median and interquartile range when distributions are skewed. Categorical variables will be summarized as frequencies and percentages. Baseline characteristics will be described across the four randomized groups. Formal hypothesis testing of baseline differences will not be used to determine covariate inclusion because treatment allocation is randomized.

The trial follows a 2 × 2 factorial design with acupuncture (yes vs no) and moxibustion (yes vs no) as the two randomized intervention factors. The principal factorial analyses will estimate the marginal main effect of acupuncture and the marginal main effect of moxibustion. The acupuncture × moxibustion interaction will also be estimated to assess whether the effect of one intervention differs according to the presence of the other intervention.

For the primary outcome, week-12 AFC will be analyzed using factorial analysis of covariance, with baseline AFC, acupuncture allocation, moxibustion allocation, the acupuncture × moxibustion interaction, and study center included in the model. The principal treatment contrasts will be the adjusted main effects of acupuncture and moxibustion. Adjusted mean differences will be reported with 95% confidence intervals and two-sided P values. Because AFC is the single registered primary outcome, no multiplicity adjustment is required across multiple primary endpoints.

Secondary continuous outcomes measured at baseline and week 12, including AMH, FSH, LH, E2, PSV, RI, PI, modified Kupperman score, SAS score, and TCM syndrome score, will be analyzed using analogous baseline-adjusted factorial models where distributional assumptions are appropriate. Secondary analyses will be interpreted as supportive rather than confirmatory. *Post hoc* pairwise comparisons among the four individual treatment groups, when performed, will be secondary to the prespecified factorial analyses and will use an appropriate multiplicity adjustment.

The acupuncture × moxibustion interaction was not used as the basis for sample-size determination and will therefore be interpreted as exploratory. Interaction estimates will be reported with 95% confidence intervals and will not be interpreted solely according to statistical significance. If a clinically important interaction is suggested, results for the four individual treatment combinations will additionally be presented to facilitate interpretation.

Clinical pregnancy will be analyzed as an exploratory reproductive outcome. Cumulative pregnancy counts and proportions will be summarized by randomized group through week 48. Subsequent ART exposure and type will be documented where available and considered in interpretation; spontaneous conceptions and conceptions following ART will be summarized separately where possible. Because ART is initiated after randomization, these subgroup summaries will not be interpreted as randomized causal comparisons.

Missing data will not be replaced using last-observation-carried-forward. The amount, pattern, and reasons for missing data will be summarized by randomized group. For the primary AFC analysis, multiple imputation under a missing-at-random assumption will be used to assess the robustness of the ITT results when week-12 data are missing; the imputation model will include randomized treatment factors, study center, baseline AFC, and other relevant baseline variables. Available-case and PP analyses will be used as additional sensitivity analyses.

Categorical outcomes will be summarized using frequencies and percentages and analyzed using the chi-square test or Fisher’s exact test where appropriate. Adverse-event incidence will be summarized by randomized treatment group and by investigator-assessed relatedness. Unless otherwise specified for *post hoc* multiple comparisons, statistical tests will be two-sided with a nominal significance level of 0.05. Effect estimates and 95% confidence intervals will be emphasized alongside P values.

### Data monitoring committee

An independent Data Monitoring Committee (DMC), comprising clinical experts, a statistician, and an ethics representative independent of the investigative team, will oversee participant safety and trial conduct. The DMC will review recruitment progress, adherence, protocol deviations, missing data, and adverse events every 3 months or as needed. No formal interim efficacy analysis or numerical stopping boundary is planned. The DMC may recommend protocol modification, temporary suspension, or early termination if unexpected safety concerns arise or if major feasibility problems are judged to preclude safe or valid completion of the trial. Any recommendation will be documented and handled in accordance with ethics requirements.

### Ethics statement

This study has been approved by the Medical Ethics Committee of the First Affiliated Hospital of Guizhou University of Traditional Chinese Medicine (Approval No. KH2024-002). The study will be conducted in accordance with local regulations, institutional requirements, and the Declaration of Helsinki. Written informed consent will be obtained from all participants before enrollment. Participants may withdraw from the study at any time without affecting their routine clinical care. The protocol was registered with the International Traditional Medicine Clinical Trial Registry (ITMCTR2025002408).

Any important protocol amendment will be submitted to the ethics committee and trial registry before implementation. The study findings will be disseminated through peer-reviewed journals, academic conferences, and clinical communication platforms. Participant-level identifiable data will not be disclosed. The participating centers will comply with the approved protocol, and local administrative permission will be obtained where required.

## Discussion

Despite growing interest in integrative approaches for premature ovarian insufficiency (POI), robust evidence supporting acupuncture and moxibustion as adjuncts to standard hormone replacement therapy (HRT) remains limited. HRT remains the cornerstone of management for women with POI who have no contraindications (10), and contemporary reviews emphasize its role in controlling hypoestrogenic symptoms and reducing long-term consequences of estrogen deficiency (7). At the same time, HRT does not replenish the depleted follicular pool or reliably restore reproductive potential (33). For women who wish to conceive using autologous oocytes, limited residual follicular activity and poor ovarian responsiveness remain major clinical challenges (12). The present trial is therefore designed to evaluate whether acupuncture, moxibustion, or their combination provides additional effects on ovarian reserve markers and related clinical outcomes when added to standard estradiol/dydrogesterone therapy.

An important methodological feature of this trial is its 2 × 2 factorial design. Previous randomized and evidence-synthesis literature has focused mainly on acupuncture as a single modality (14), and a pilot clinical study likewise evaluated acupuncture without separately estimating a moxibustion effect (18). Broader reviews of complementary approaches to POI describe acupuncture, moxibustion, and related interventions but do not resolve their independent contributions (13). Clinical work combining acupuncture with Western medicine has also been reported (23), while randomized acupuncture protocols for diminished ovarian reserve generally use conventional parallel-group comparisons (25). By treating acupuncture and moxibustion as two separate randomized factors, the present design permits estimation of the main effect of each intervention within the same trial and exploratory assessment of whether the effect of one modality differs according to the presence of the other. This is clinically relevant because the two modalities may be used separately or together. However, because the study was not specifically powered for the acupuncture × moxibustion interaction, any apparent synergistic or antagonistic effect will be regarded as hypothesis-generating and interpreted primarily through effect estimates and confidence intervals.

From a biomedical perspective, several mechanisms have been proposed through which acupuncture might influence ovarian function. Mechanistic literature suggests that acupuncture may modulate hypothalamic-pituitary-ovarian regulation, autonomic activity, neuroendocrine responses, and pelvic or ovarian blood perfusion (17). A pilot clinical study in premature ovarian failure also reported changes in endocrine and symptom-related measures after acupuncture (18). Moxibustion provides a different form of stimulation, predominantly through local thermal exposure. Experimental work in diminished ovarian reserve has linked moxibustion with oxidative-stress-related pathways and ovarian tissue responses (20). Separate animal evidence suggests effects on granulosa-cell apoptosis and PI3K/AKT signaling (21). These observations provide biological hypotheses relevant to residual ovarian activity and the ovarian microenvironment in POI, but they should not be interpreted as established mechanisms of clinical efficacy in women with POI. The present trial is designed primarily to evaluate clinical outcomes rather than to provide direct mechanistic validation.

Separately, the intervention protocol was informed by the theoretical framework of Traditional Chinese Medicine (TCM). TCM-based POI literature commonly describes kidney deficiency and blood stasis among the syndrome patterns considered in treatment planning (16). The acupoint prescription used in this trial was selected according to these traditional principles and the clinical treatment framework adopted by the study team. A complex-network analysis of acupuncture and moxibustion prescriptions for diminished ovarian reserve and premature ovarian failure identified CV4, SP6, ST36, BL23, and EX-CA1 among frequently used points (22). A previous clinical study of POI also used a Ren-regulating and kidney-tonifying acupuncture approach in combination with Western medicine (23). The same predefined acupoint prescription is used across the acupuncture, moxibustion, and combined-treatment groups to maintain a consistent treatment framework while varying the mode of stimulation. These TCM concepts are presented as the traditional rationale for treatment selection and should be distinguished from contemporary biomedical mechanisms.

Another strength of the study is the use of multidimensional outcome measures with a prespecified hierarchy. Antral follicle count (AFC) is the registered primary outcome and provides an ultrasound-based estimate of the recruitable antral follicle pool, a clinically recognized component of ovarian reserve assessment in POI (7). AFC has also been used as an endpoint in acupuncture trials included in the POI evidence base (14). Anti-Müllerian hormone (AMH) is a secondary ovarian reserve marker that provides complementary information on residual follicular activity. Serum follicle-stimulating hormone (FSH), luteinizing hormone (LH), and estradiol (E2) provide supportive information regarding endocrine status, while ovarian Doppler parameters provide secondary information on ovarian perfusion. The modified Kupperman Scale captures hypoestrogenic symptom burden, whereas the Self-Rating Anxiety Scale (SAS) addresses psychological distress, which is frequently reported in women with POI (2). The week-12 endpoint is intended as a prespecified short-term biomarker assessment window rather than a threshold at which restoration of ovarian reserve is expected. This interpretation is supported by POI-specific clinical data showing measurable AFC and AMH changes after a three-month treatment regimen (23) and by a recent 12-week sham-controlled DOR trial in which the between-group improvement in AFC was significant whereas the between-group AMH difference was not (24). Importantly, neither AFC nor AMH is a direct measure of fertility. Improvement in these markers should not be interpreted as evidence of replenishment of the primordial follicle pool, improved oocyte competence, spontaneous conception, or increased live-birth probability. Clinical pregnancy is therefore treated as an exploratory reproductive outcome; the study was not powered for pregnancy endpoints, and conception during follow-up may be strongly influenced by subsequent ovulation induction, *in vitro* fertilization and embryo transfer (IVF-ET), or other assisted reproductive technology (ART).

Several limitations should be considered. First, because acupuncture and moxibustion are physical interventions, participants and treatment practitioners cannot be blinded. Although blinded outcome assessment, blinded laboratory evaluation, blinded statistical analysis, and standardized intervention procedures are intended to reduce detection and analytical bias, performance bias cannot be completely excluded. Second, the trial does not include sham acupuncture or sham moxibustion controls. Consequently, particularly for subjective outcomes such as menopausal symptoms, anxiety, and TCM syndrome scores, observed differences may reflect not only intervention-specific effects but also treatment expectations, attention from practitioners, therapeutic interaction, repeated contact with the clinical team, and other contextual or placebo-related effects. The absence of a sham comparator therefore prevents the trial from fully separating the specific physiological effects of acupuncture or moxibustion from nonspecific treatment and contextual effects. More objective outcomes such as AFC and AMH are less directly susceptible to expectation effects, but indirect influences through adherence, health behaviors, concomitant care, or differential participant engagement cannot be completely excluded. Moreover, designing truly inert sham procedures for acupuncture and moxibustion is methodologically challenging because sham needling or thermal stimulation may itself produce sensory or physiological effects. Future studies using carefully validated sham or attention-control procedures may help distinguish specific and contextual components of treatment response.

Third, the study was not powered specifically for the acupuncture × moxibustion interaction or for pregnancy outcomes. Interaction and reproductive analyses will therefore be considered exploratory and should not be used to draw definitive conclusions regarding synergistic treatment effects or fertility improvement. Fourth, the study population represents a selected subgroup of women with POI rather than the broader POI population: participants are aged 22–40 years, have reproductive needs, meet the prespecified FSH range, and are recruited during the pretreatment period before assisted reproduction. POI itself is clinically heterogeneous in etiology, severity, residual ovarian activity, and reproductive prognosis (33); therefore, the present eligibility criteria may limit generalizability to adolescents, women with more advanced ovarian failure, different etiologies, no fertility intentions, women outside the prespecified FSH range, or those with substantial comorbidity. Existing acupuncture evidence also includes heterogeneous populations and study designs (14), reinforcing the need for caution when extrapolating results beyond the present eligibility criteria. Fifth, subsequent fertility treatments during follow-up may differ between participants and may influence pregnancy outcomes independently of randomized treatment assignment. Finally, the week-24 and week-48 visits focus mainly on reproductive follow-up rather than repeated measurement of ovarian reserve and endocrine biomarkers; consequently, the protocol will not establish the long-term durability of any week-12 biomarker changes.

Notwithstanding these limitations, this multicenter factorial trial may provide useful randomized evidence regarding the independent adjunctive effects of acupuncture and moxibustion in women with POI receiving standard HRT. By separating the two intervention factors while maintaining a common HRT background and standardized acupoint framework, the study may clarify whether either modality is associated with changes in the primary AFC outcome and supportive secondary clinical outcomes and may generate preliminary evidence regarding their potential interaction. The findings should be interpreted in terms of changes in ovarian reserve markers and related clinical outcomes rather than restoration of fertility. They may also provide a methodological basis for future larger trials designed specifically to evaluate interaction effects and clinically definitive reproductive outcomes such as live birth.
